# Supplementary material for: Comparison of Glycemic Variability and Hypoglycemic Events in Hospitalized Older Adults Treated with Basal Insulin plus Vildagliptin and Basal–Bolus Insulin Regimen: A Prospective Randomized Study
Source: J Clin Med. 2022 May 16;11(10):2813. doi: 10.3390/jcm11102813 (PMC9143484; doi:10.3390/jcm11102813)
Supplement: Supplementary file 1 [file jcm-11-02813-s001.zip › jcm-1702141-supplementary.pdf]

Supplementary data

Table S1. Treatment regime glargine-DPP4i group.

Patients treated with diet or only one oral antidiabetic agent<br>- CBG 150-250 mg/dl: 0.2 UI/Kg/d<br>- CBG >250 mg/dl: 0.3 UI/Kg/d
Patients treated with more than one oral antidiabetic agent<br>- CBG <150 mg/dl: 0.2 UI/Kg/d<br>- CBG 150-250 mg/dl: 0.3 UI/Kg/d<br>- CBG >250 mg/dl: 0.4 UI/Kg/d
Patients treated with basal insulin +/- any oral antidiabetic<br>- CBG <150 mg/dl: 0.3 UI/Kg/d<br>- CBG 150-250 mg/dl: 0.4 UI/Kg/d<br>- CBG >250 mg/dl: 0.5 UI/Kg/d
Patients treated with ≥ two insulin dose +/- any oral antidiabetic<br>- Any CBG: 0,6 UI/Kg/d
**Daily insulin adjustment**
Fasting and pre-meal BG between 100-180 mg/dl without hypoglycemia the previous day: same dose
Fasting and pre-meal BG >180 mg/dl: increase glargine dose by 10%
Fasting and pre-meal BG between 70-99 mg/dl: decrease glargine dose by 10%
If hypoglycemia (<70 mg/dL): decrease glargine dose by 20%
If hypoglycemia (<40 mg/dL): decrease glargine dose by 30-40%.
**Supplemental (correction) insulin:**
If CBG was >300 mg/dL, 6 UI of aspart insulin was administered

*Abbreviations: BG, blood glucose; CBG, capillary blood glucose.

Table S2. Treatment regim basal-bolus group.

Patients treated with diet or only one oral antidiabetic agent<br>- CBG 150-250 mg/dl: 0.3 UI/Kg/d<br>- CBG >250 mg/dl: 0.4 UI/Kg/d
Patients treated with more than one oral antidiabetic agent<br>- CBG <150 mg/dl: 0.3 UI/Kg/d<br>- CBG 150-250 mg/dl: 0.4 UI/Kg/d<br>- CBG >250 mg/dl: 0.5 UI/Kg/d
Patients treated with basal insulin +/- any oral antidiabetic<br>- CBG <150 mg/dl: 0.4 UI/Kg/d<br>- CBG 150-250 mg/dl: 0.5 UI/Kg/d<br>- CBG >250 mg/dl: 0.6 UI/Kg/d
Patients treated with ≥ two insulin dose +/- any oral antidiabetic<br>- Any CBG: 0,6 UI/Kg/d
Half of total daily dose will be given as glargine and half as aspart

**Daily glargine insulin adjustment**
Fasting and pre-meal BG between 100-180 mg/dl without hypoglycemia the previous day: no change
Fasting and pre-meal BG >180 mg/dl: increase glargine dose by 20%
Fasting and pre-meal BG between 70-99 mg/dl: decrease glargine dose by 10%
If hypoglycemia (BG <70 mg/dL): decrease glargine dose by 20%
If hypoglycemia (BG <40 mg/dL): decrease glargine dose by 30-40%.
**Daily aspart insulin adjustment**
Increase aspart insulin dose following the scale (supplementary table 2.1) for BG >140 mg/dl based on TDI dose
Decrease aspart insulin dose following the scale (supplementary table 2.1) for BG <80 mg/dl based on TDI dose

*Abbreviations: BG, blood glucose; CBG, capillary blood glucose; TDI, total daily insulin.

Table S3. Aspart insulin scale.

BG (mg/dL)	TDI <40 UI	TDI 40-80 UI	TDI >80 UI
**<80**	-2	-2	-2
**81-140**	+0	+0	+0
**141-190**	+1	+1	+2
**191-240**	+2	+3	+5
**241-290**	+4	+6	+7
**291-350**	+6	+9	+10
**>350**	+7	+11	+12

*Abbreviations: BG, blood glucose; TDI, total daily insulin.
